# Social-Stress Disorder and Post-Traumatic Stress Disorder in Ukrainian Population.

**DOI:** 10.1192/j.eurpsy.2023.1920

**Published:** 2023-07-19

**Authors:** O. M. Pityk

**Affiliations:** Psychiatry, Narcology and Medical Psychology, Ivano-Frankivsk National Medical University, Ivano-Frankivsk, Ukraine

## Abstract

**Introduction:**

The situation that has been going on in Ukraine for the past six months has led to catastrophic consequences. This forces neuroscientists and, including psychiatrists, to turn to the study of the impact of all these events on the mental health of the population of Ukraine. In 90th of XX Russian psychiatrist Y. A. Alexandrovsky expressed opinion of presence the group of so-called social-stress disorders (SSD) that was determined like psychogenic-actual for most people in definite social, economic and political situation. Most of the people in Ukraine now experience both SSD and post-traumatic stress disorder (PTSD).

**Objectives:**

Most of the people in Ukraine now experience both SSD and post-traumatic stress disorder (PTSD).

**Methods:**

The method of clinic-psychopathological interview with patients who applied out-patient consultation on the chair of psychiatry.

**Results:**

The main changes in psychic state include following behaviors and clinical implications: loss of the value of human life, which is manifested in indifference to death in lowering caution when hazardous situations, willingness to sacrifice lives without any ideals. There is unrestrained lost for pleasure and moral promiscuity, exacerbation of personality typological traits, development of hyperstenic reactions (to self-destructive non-expedient behavior), hypostenic disorders, panic reactions, depression, dissociative and conversive irregularities, loss of communicational plasticity, loss of the ability to adapt to what happens with the preservation prospects of targeted actions, manifestations of cynicism, the tendency to antisocial actions. Patients had complaints on increase anxiety, pessimistic attitudes, existential vacuum, sense of uselessness and loss of perspectives, tendency to irrational perception of reality with including mechanisms of autistic and archaic thinking.

**Conclusions:**

Thus, psychological status of the population of Ukraine is a model of complicated combination of SSD plus PTSD can be considered like a basis which leads to the decreasing of the individual barrier of mental adaptation with the next manifestation of different forms of psychopathological syndromes and needs further in-depth and detailed research.

**Disclosure of Interest:**

None Declared

